# Letter regarding ‘early intervention with rescue ERCP and pancreatic stenting for unanticipated post-ERCP pancreatitis: a comparative study’

**DOI:** 10.1080/07853890.2026.2639717

**Published:** 2026-03-12

**Authors:** ibrahim Ethem Güven

**Affiliations:** Department of Gastroenterology, Yenimahalle Training and Research Hospital, Ankara, Turkey

Dear Editor

I read with great interest the study conducted by An et al. [1] evaluating the effectiveness of early rescue ERCP and pancreatic stenting for unanticipated post-ERCP pancreatitis.

The most common complication of ERCP is post-ERCP pancreatitis (PEP). During ERCP procedure, pancreatic stenting is a well-known and frequently used method in cases of difficult cannulation, unintended cannulation of the pancreatic duct, and in high-risk patients [2]. However, treatment options in unanticipated PEP are still controversial [3]. In this context, the authors present significant data indicating that early rescue ERCP with pancreatic duct stenting may improve clinical outcomes in selected patients with unanticipated PEP.

The reduction in pain intensity, duration of systemic inflammatory response syndrome, and time to oral intake observed in the rescue ERCP group are clinically significant outcomes. These findings suggest that rescue ERCP and pancreatic stenting may reduce disease progression and severity by lowering pancreatic duct pressure. To further clarify the clinical implications of these findings, I would like to offer several observations and important considerations.

Firstly, the retrospective design and limited sample size are insufficient to support definitive clinical conclusions, and prospective studies with larger sample sizes are required to support this hypothesis. Second, although no procedure-related complications were observed in this cohort, the sample size may be insufficient to detect rare adverse events. Since current guideline recommendations do not routinely support rescue pancreatic stenting in established PEP [4], more high-quality evidence is required to better define the risk-benefit profile of this approach. Third, the determination of an approximately 7-hour therapeutic window was based on subgroup analyses involving a very limited number of patients, and the conclusions drawn may therefore be prone to statistical instability. Lastly, the non-use of rectal NSAIDs in this cohort, which are recommended in current guidelines [5], may have affected the study results by influencing the incidence and severity of PEP.

Despite these limitations, this study constitutes a notable and commendable contribution to the literature. The authors should be congratulated for addressing an important clinical gap in the management of unanticipated PEP. The current study may provide a valuable foundation for future studies that could personalize the treatment of unanticipated PEP.

## Data Availability

Data sharing does not apply to this article, as no data were created or analysed in this study.
